# Study of peripheral dose from low-dose CT to adaptive radiotherapy of postoperative prostate cancer

**DOI:** 10.3389/fonc.2023.1227946

**Published:** 2023-11-01

**Authors:** Guanghui Gan, Wei Gong, Lecheng Jia, Wei Zhang, Shimei Wang, Juying Zhou, Hua Jiang

**Affiliations:** ^1^ Department of Radiation Oncology, First Affiliated Hospital of Soochow University, Suzhou, China; ^2^ Real-time Lab, Shenzhen United Imaging Research Institute of Innovative Medical Equipment, Shenzhen, China; ^3^ Zhejiang Engineering Research Center for Innovation and Application of Intelligent Radiotherapy Technology, Wenzhou, China; ^4^ Radiotherapy Business Unit, Shanghai United Imaging Healthcare Co., Ltd., Shanghai, China; ^5^ Central Research Institute, United Imaging Healthcare Group, Shanghai, China

**Keywords:** peripheral dose, adaptive radiotherapy, low-dose CT, deep learning, prostate cancer

## Abstract

**Objectives:**

The increasing use of computed tomography (CT) for adaptive radiotherapy (ART) has raised concerns about the peripheral radiation dose. This study investigates the feasibility of low-dose CT (LDCT) for postoperative prostate cancer ART to reduce the peripheral radiation dose, and evaluates the peripheral radiation dose of different imaging techniques and propose an image enhancement method based on deep learning for LDCT.

**Materials and methods:**

A linear accelerator integrated with a 16-slice fan-beam CT from UIH (United Imaging Healthcare, China) was utilized for prostate cancer ART. To reduce the tube current of CT for ART, LDCT was acquired. Peripheral doses of normal-dose CT (NDCT), LDCT, and mega-voltage computed tomography (MV-CT) were measured using a cylindrical Virtual Water™ phantom and an ion chamber. A deep learning model of LDCT for abdominal and pelvic-based cycle-consistent generative adversarial network was employed to enhance the image quality of LDCT. Six postoperative prostate cancer patients were selected to evaluate the feasibility of low-dose CT network restoration images (RCT) by the deep learning model for ART. The three aspects among NDCT, LDCT, and RCT were compared: the Hounsfield Unit (HU) of the tissue, the Dice Similarity Coefficient (DSC) criterion of target and organ, and dose calculation differences.

**Results:**

In terms of peripheral dose, the LDCT had a surface measurement point dose of approximately 1.85 mGy at the scanning field, while the doses of NDCT and MV-CT were higher at 22.85 mGy and 29.97 mGy, respectively. However, the image quality of LDCT was worse than NDCT. When compared to LDCT, the tissue HU value of RCT showed a significant improvement and was closer to that of NDCT. The DSC results for target CTV between RCT and NDCT were also impressive, reaching up to 94% for bladder and femoral heads, 98% for rectum, and 94% for the target organ. Additionally, the dose calculation differences for the ART plan based on LDCT and NDCT were all within 1%. Overall, these findings suggest that RCT can provide an effective alternative to NDCT and MV-CT with similar or better outcomes in HU values of tissue and organ damage. More testing is required before clinical application.

## Introduction

At present, radiotherapy is one of the main methods for postoperative prostate cancer treatment. The intensity modulated radiotherapy (IMRT) and volumetric-modulated arc therapy (VMAT) are commonly used techniques for prostate cancer due to their highly conformal dose distributions and dose homogeneity. However, the highly conformal dose distributions of the technology bring an important problem that needs to be solved: the application of the above technique is the ideal result under the condition that the tumor target is a stationary rigid body, as radiotherapy was fractional delivered. In the subsequent fractions, the position and shape of targets and organs change greatly due to the rectal peristalsis and vesical fulling, and make the targets deviate from the radiation field, resulting in underdose of tumors or overdose of organs. In addition to the setup errors, the position of the actual target and planned target changed. Therefore, the most commonly used method in clinical practice is to add margins by expanding the clinical target volume (CTV) to the planning target volume (PTV). The margins are generally over 6–8 mm according to different immobilization methods and weight change of the patient. The position change of organs is an important reason for the unsatisfactory effect of abdominal tumor radiotherapy. For the era of image-guided radiotherapy (IGRT), this is a difficult and hot spot that has been paid more and more attention (1, 2). The medical images acquired by different devices were spatially transformed with the planning CT for radiotherapy, and based on image registration technology, the region of interest (ROI) of the images can be strictly matched to calibrate setup errors among radiotherapy fractions (3–5). However, IGRT cannot completely correct the position deviation of the target due to rectal peristalsis, vesical fulling, and intestinal gas. Online adaptive radiotherapy (ART) technology is an effective approach to correct for morphological changes and setup errors in a patient’s anatomy (6). In the process of radiotherapy, ART uses different medical images, such as CT, magnetic resonance (MR), or kilo-voltage cone beam CT (CBCT) performed in the treatment room to reassess current position and shape of target volumes and normal anatomy (7, 8). The target and normal tissues were delineated according to the online images, and the radiotherapy plan was adjusted to reduce the dose to normal organs. Compared with CT, MR images cannot provide the electron density information for dose calculation during the treatment planning process (9). The CBCT images cannot provide clear soft-tissue contrast images with obvious artifacts due to x-ray scattering and noise (10).

Daily CT is being implemented for prostate cancer radiotherapy due to the deformation of the bladder and the rectum. This naturally leads to the problem of image dose and then to the issue of peripheral dose. The peripheral dose delivered to the patient is dependent on the number of ART. The dose within the imaged area for CBCT used for radiation therapy is well reported, and their measurements of patient dose have been published. The typical dose of the isocenter for a single CBCT is 1.5–3.0 cGy (11–15). The risk of cancer induced by ionizing radiation has been confirmed, and the risk of a secondary cancer is greatly increased by radiotherapy. Therefore, how to implement ART with the lowest peripheral dose is an urgent problem to be solved.

Low-dose CT (LDCT) was first proposed by Naidich et al. in 1990, which was defined as a new idea of keeping other scanning parameters unchanged and appropriately reducing the tube current to meet the diagnostic requirements and reduce the radiation dose to the patients (16, 17). It leads to a decrease in the number of photons received by the detector, and the projection data are polluted by noise (18). The reconstructed CT images have a lot of noise and artifacts, which affect the identification of anatomical structures (16). At present, cycle-consistent generative adversarial network (CycleGAN) is widely used in the conversion of different modal images (19–21). The CycleGAN adopts a dual-generator network structure and a cycle-consistent loss function to achieve network parameter training, which has become a commonly used network structure in LDCT image denoising processing (22, 23). Obviously, the implementation of ART based on low-dose CT after image denoising can reduce the peripheral dose. Furthermore, there are no studies evaluating the peripheral dose for ART based on LDCT.

In this study, a cylindrical Virtual Water™ phantom and ion chamber were used to measure the dose of LDCT, NDCT, and mega-voltage computed tomography (MV-CT). An image enhancement model for the abdomen and pelvis based on a Content-Noise Cycle-Consistent Generative Adversarial Network (CNCycle-GAN) was used to improve the quality of LDCT to low-dose CT network restoration images (RCT). Then, the objective evaluation parameters of the images and automatic delineation performance were used to evaluate whether RCT met the ART requirements. The purpose of this study was to assess the amount of peripheral dose of LDCT and evaluate the long-term results of the new technique on patients using ART.

## Materials and methods

### Peripheral dose measurement

**Figure 1:**
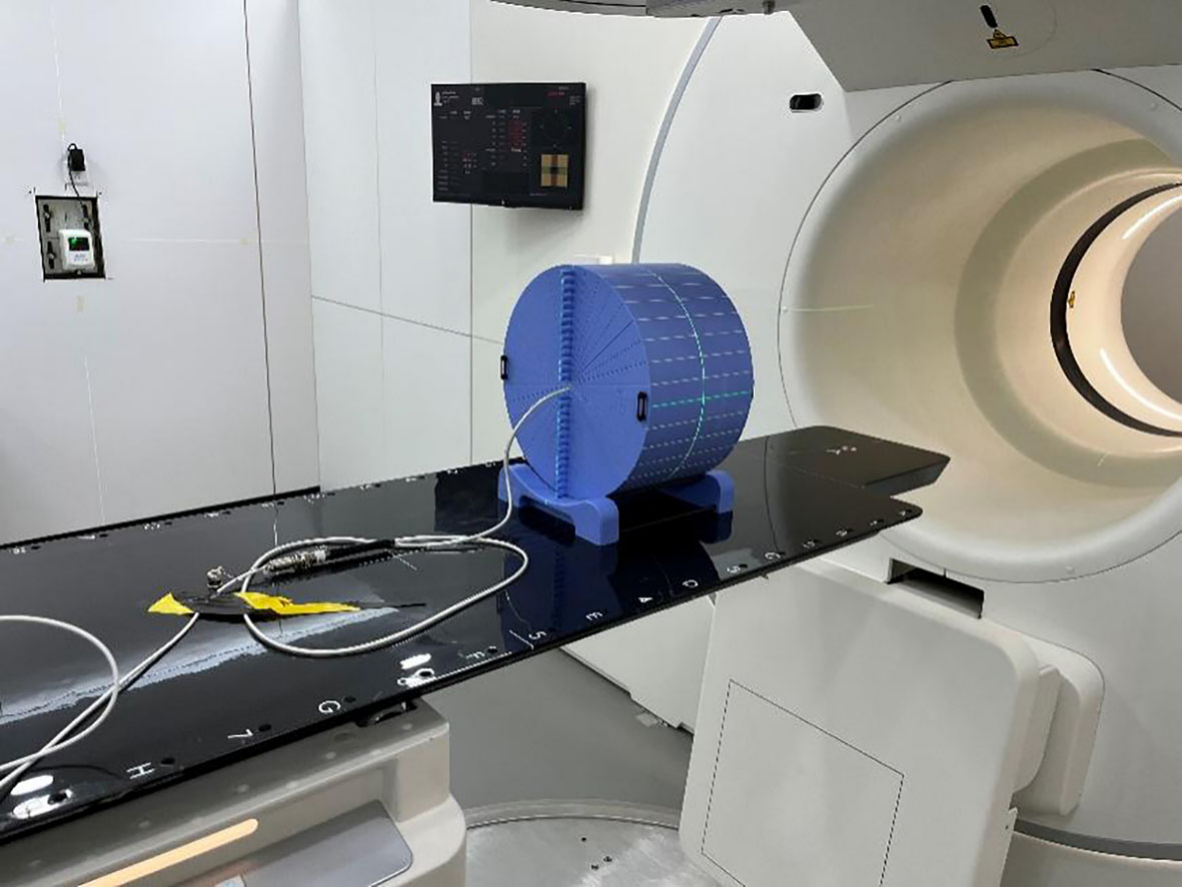
The cylindrical Virtual Water™ phantom in place on the treatment couch.

The dose delivered to a patient receiving LDCT, NDCT, or MV-CT was determined by irradiating a cylindrical Virtual Water™ phantom (**Figure 1**). In the central axis, the cylindrical phantom contains 29 Virtual Water™ plugs that can be removed for ion chamber insertion. The length and diameter of the phantom are 30 cm, and the image scan length was selected to be 15 cm; the central plane was chosen at the center of the phantom. The peripheral dose measurements of NDCT and LDCT were performed on an uRT-linac 506c. This machine integrates a 16-slice helical CT to acquire diagnostic-grade FBCT. The dose of MV-CT was measured with an Accuray TomoTherapy.

The Exradin A1SLMR Ion Chamber was selected to load into the measurement circle of the phantom to measure the dose. The collecting volume of the ion chamber is 0.053 cm^3^, and the collector diameter is 1.0 mm. The ion chamber has been calibrated by x-ray in the University of Wisconsin-Madison Dosimetry Calibration Laboratory. The beam quality of x-ray generally is described by a half-value layer (HVL), and the HVL of the x-ray used to calibrate the ion chamber is 6.77 mm Al.

Along the vertical central axis, the ion chamber was placed at 0.5 cm (distance from center) and then at 1.5 cm, 2.5 cm, 4.5 cm, 8.5 cm, 12.5 cm, and 14.5 cm, the distance of the last point was 5 mm from surface, and all measurement points were on the central (internal) plane of the phantom. Along the long axis, the ion chamber was placed at 0 cm (distance from center) and then at 1 cm, 2 cm, 4.5 cm, 7.5 cm, 13.5 cm, and 19.5 cm, and both measurement points were positioned at 5 mm distance from the surface. This arrangement enables the measurement of radiation doses in and out of the field.

**Figure 2:**
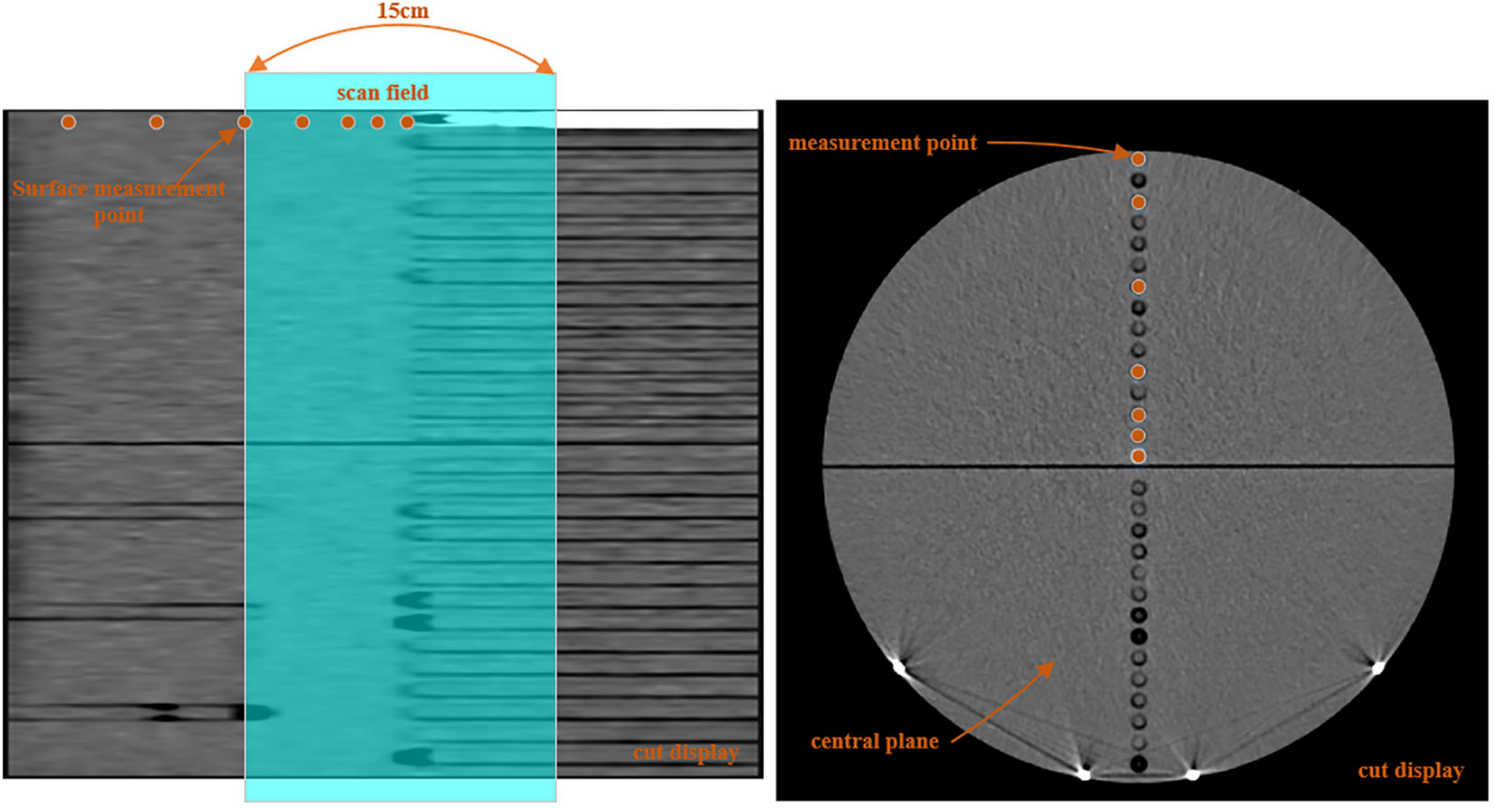
Schematic of the dose measurement positions in versions of the phantom, with the shade area representing the CT scan field.

**Figure 2** shows a lateral and transversal view of the phantom and the image length by a 15-cm-long CT scan. The NDCT used to derive the peripheral doses was a clinically validated protocol for pelvis, the tube voltage was 120 kV, and the tube current was 233 mA. The protocol takes a helical scan and the slice thickness is 3 mm. The LDCT scan conditions were consistent with the NDCT except for the tube current; the tube current of LDCT was 23 mA to be consistent with the scanning current of the network model. In theory, the dose distributions of NDCT and LDCT should follow a scaling factor of 10 (233 mA/23 mA). The same scan length was used on a helical scan of MV-CT to measure the peripheral dose. The scanning protocols for abdominal patients were used.

### LDCT enhancement method

As for LDCT enhancement for patients in this study, the uRT-linac 506c accelerator from UIH was used to acquire LDCT to achieve IGRT for prostate cancer. The image enhancement model based deep learning for abdominal and pelvic region was selected (24). The images of 76 patients with abdominal and pelvic tumors who received radiotherapy were selected, and each patient underwent two helical scans with normal dose (tube voltage, 120 kV; tube current, 233 mA) and low dose (tube voltage, 120 kV; tube current, 24 mA) in IGRT, and then the training model was established based on the image data and CNCycle-GAN network.

**Figure 3:**
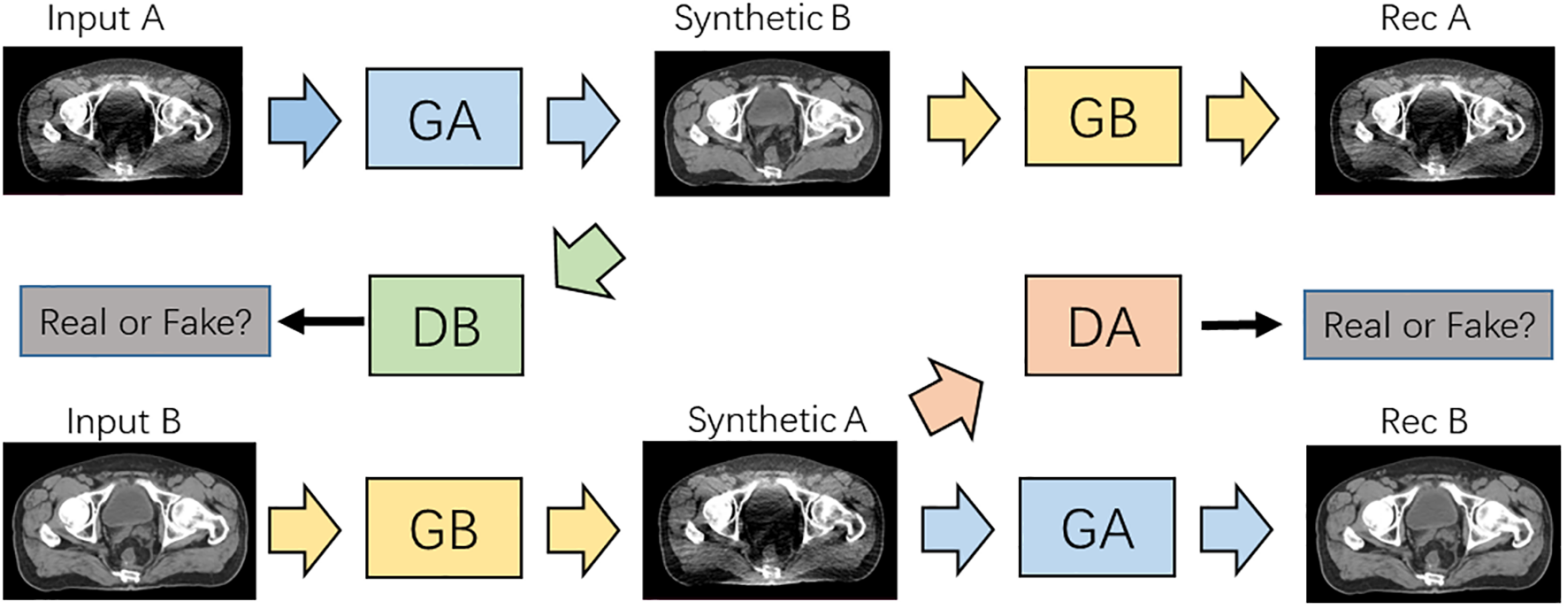
Architecture of the CNCycle-GAN network.

As shown in **Figure 3**, the CNCycle-GAN network was proposed to achieve bidirectional conversion between two domain images (A, LDCT; B, NDCT). Different from CycleGAN, CNCycle-GAN uses two content noise convolutional networks as generators, *G_A_*:A→B, to convert LDCT to NDCT, *G_B_*:B→A, to convert NDCT to LDCT. Each generator is adversarially trained *D_B_* and *D_A_* with the corresponding convolutional network discriminator.

### Network training

For network training, we partitioned the NDCT and LDCT images of 76 patients for network parameter optimization. Prior to optimizing network parameters, we performed data preprocessing by normalizing CT image pixel values layer-by-layer within the range of (0, 1) based on preset window width (WW) and window level (WL), thereby enhancing model training efficiency. We set the WL at 400 HU and the WW at 2,800 HU. To improve the network robustness and reduce memory limitations during training, we randomly cropped normalized images into image blocks of 256256 size, and applied data augmentation techniques such as random rotation and flip.

During the network training phase, we utilized the Adam optimization method to train the CNCycle-GAN network. The training duration was set to 250 epochs. There were two stages implemented for controlling the learning rate during training: initially, the learning rate was set to 0.0002 for the first 150 epochs and subsequently linearly decreased to zero in subsequent epochs. The mini-batch size was set as 4. Prior to training, network parameters were initialized with values generated from a standard normal distribution. We use PyTorch architecture to implement the CNCycle-GAN network, and using NVIDIA Quadro RTX 4000 GPUs to train the network parameters.

### Evaluation method

We compared the peripheral dose measurement for LDCT, NDCT, and MV-CT of the central axis and along the long axis of the measurement phantom. The lower peripheral dose was shown to be safer for patients.

**Figure 4:**
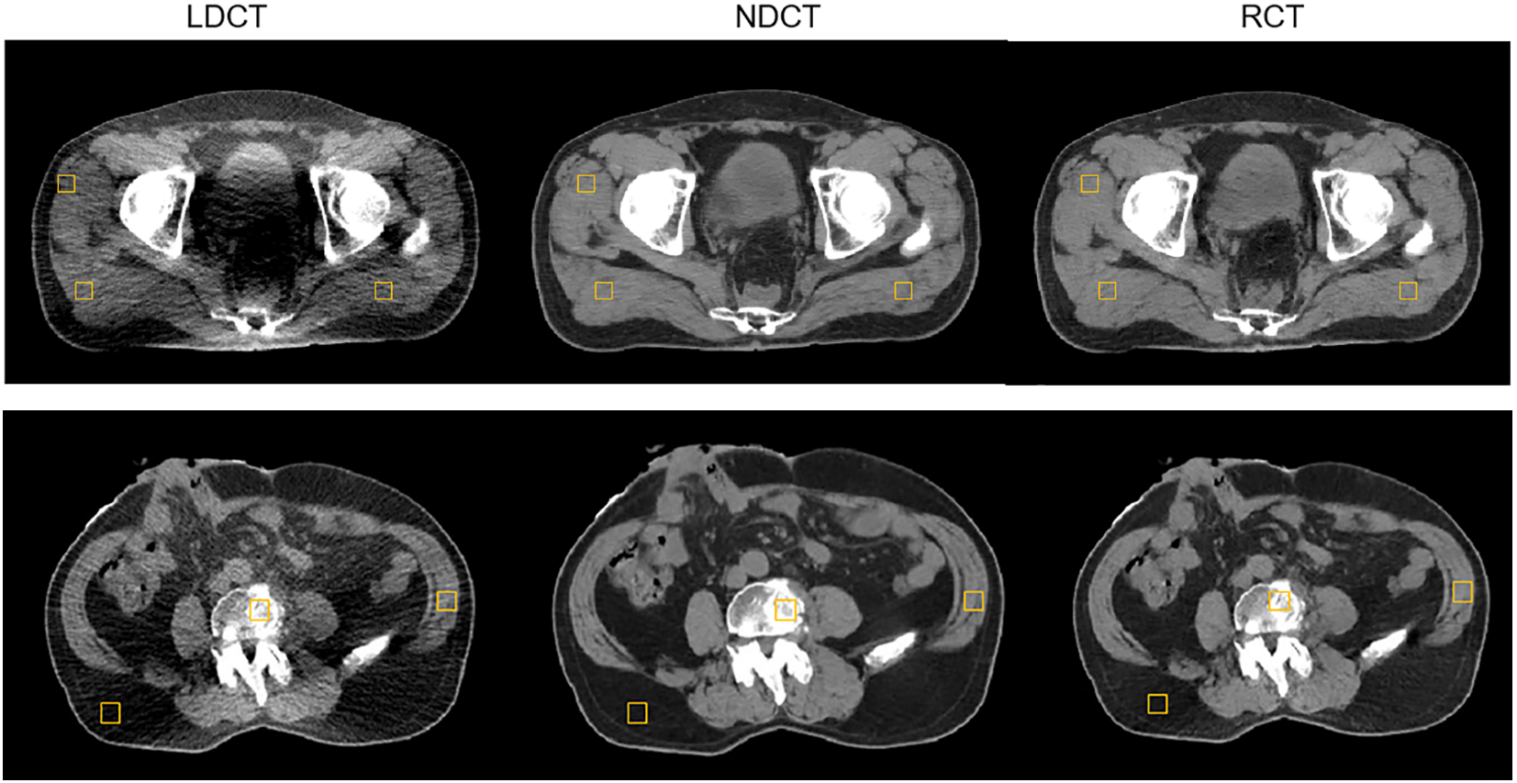
Example of evaluating the regions of interest (ROI) distribution; three muscle square ROIs were placed on a selected slice of LDCT, NDCT, and RCT for a postoperative prostate cancer test patient.

We selected six postoperative prostate cancer patients who received online-ART based on NDCT on uRT-linac 506c during March 2023; the prescription dose is 66 Gy/33 fractions to the PTV. During one fraction of adaptive radiation therapy, patients underwent a single low-dose CT helical scan at the end of the NDCT scan. This was a prospective study approved by the local institutional review board, and all patients enrolled were informed and signed consent forms before treatment. The LDCT images were restored to RCT images by the above-mentioned deep learning network model. The LDCT, RCT, and NDCT image pairs were used for the quantitative evaluation. The resulting RCT images were compared to the original LDCT and NDCT images using the HU values from four different types of tissues (muscle, fat, bladder, and bone). Three square ROIs with pixels on one slice of each patient were selected for a quantitative evaluation of the muscle (**Figure 4**) and fat regions. ROIs in each tissue were randomly selected. The positions of the ROIs are located on the regions of the same soft tissue area. Two slices at 6-mm intervals per patient were selected for the bladder and the bone, and one square ROI with pixels was positioned in the regions of the same tissue area on the selected slice.

We use the Dice Similarity Coefficient (DSC) criterion to evaluate the structure familiarity in the segmentation process. To evaluate the performance difference between the RCT and NDCT images, a senior radiation oncologist was invited to contour the CTV, the bladder, the rectum, and the femoral heads. The contouring results of RCT and NDCT were compared using DSC.

The plan (N_plan) at the fraction with ART performed on NDCT for the six cases was copied to the corresponding RCT to generate the reference plan (R_plan), and the dose of R_plan was calculated based on the same parameters. The dose distribution differences were compared between R_plan and N_plan from assessing the D_95%_, D_2%_ (dose to 95% and 2% of volume), and D_mean_ (mean dose) of the target PTV. For organ at risk (OAR) doses, the V_20 Gy_ (the percentage of volume receiving 20 Gy dose), D_2%_, and D_mean_ were compared for the rectum, the bladder, and the femoral heads.

## Results

### Peripheral dose measurement

**Figure 5:**
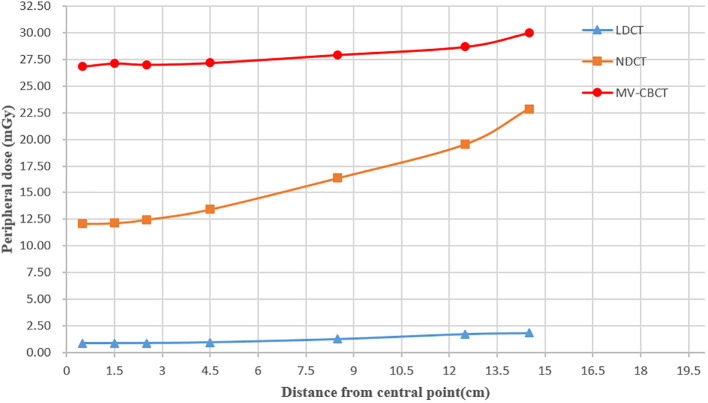
Peripheral dose measurement for LDCT, NDCT, and MV-CT in the central axis at the central plane of the measurement phantom.

For the LDCT, NDCT, and MV-CT helical scan, the peripheral dose of the central axis at the central plane of the measurement phantom is shown in **Figure 5**. The results show that the peripheral dose of MV-CT, NDCT, and LDCT to the central axis at the measurement point of surface (0.5 cm distance from surface) was 30 mGy, 22.85 mGy, and 1.85 mGy, respectively, and the dose of 0.5 cm measurement point (0.5 cm distance from central point) was 26.83 mGy, 12.1 mGy, and 0.89 mGy, respectively. In the scanning field, the closer to the surface at the central axis, the higher the dose of MV-CT, NDCT, and LDCT. The results show that the peripheral dose of MV-CT was highest at the same measurement points, and the dose of LDCT was lowest. The dose of MV-CT was approximately 27 times that of the LDCT, the NDCT was approximately 13 times. LDCT was significant in reducing radiation risk especially for daily CT scanning in prostate cancer.

**Figure 6:**
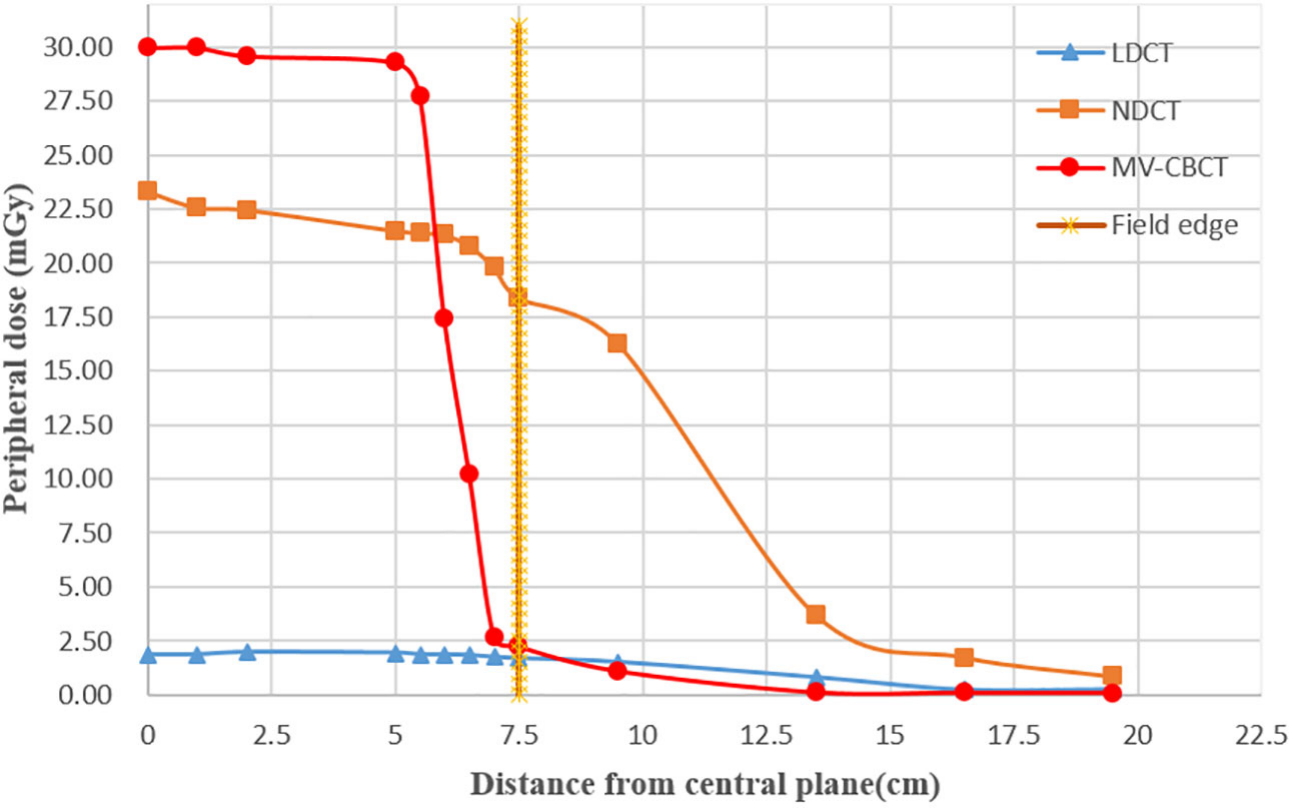
Peripheral dose measurement for LDCT, NDCT, and MV-CT in the long axis at the measurement points (5 mm distance from surface); the dotted line represents the edge of the scan field.

The peripheral doses close to 5 mm from the surface along the long axis were recorded, and results are shown in **Figure 6**. The field edge is marked at 7.5 cm from the center plane as the nominal scan length is 15 cm. By comparison, the results show that the peripheral dose of NDCT (average dose: 15.56 mGy) and that of MV-CT (27.82 mGy) were much higher than that of LDCT (1.22 mGy) in scanning field. The peripheral doses of both NDCT and LDCT began to drop off when the distance from the field edge was approximately 7.5 cm, and the doses of MV-CT began to drop off substantially at 5 cm distance from the field edge. Outside the scanning field, the farther away from the field edge, the lower the dose of the NDCT, LDCT, and MV-CT, and the dose of MV-CT was the lowest. The dose of NDCT (0.85 mGy) was higher than that of LDCT (0.26 mGy) at the same measurement point 12 cm away from the field edge. As the peripheral dose of MV-CT was higher than NDCT, there was no advantage in terms of image quality and segmentation accuracy compared to LDCT. Hence, we exclude MV-CT from the evaluations on image quality and suitability for radiation therapy.

The dose distributions of NDCT and LDCT approximately followed a scaling factor of ~10 (233 mA/23 mA), in agreement with the expectation based on theory, except for a few points. We believed that there were two possible reasons; one was the measurement error of the instrument during LDCT dose measurement. Secondly, during CT scanning, the stability of the tube current led to errors in the actual image dose.

### Deep-learning-based LDCT enhancement

**Figure 7:**
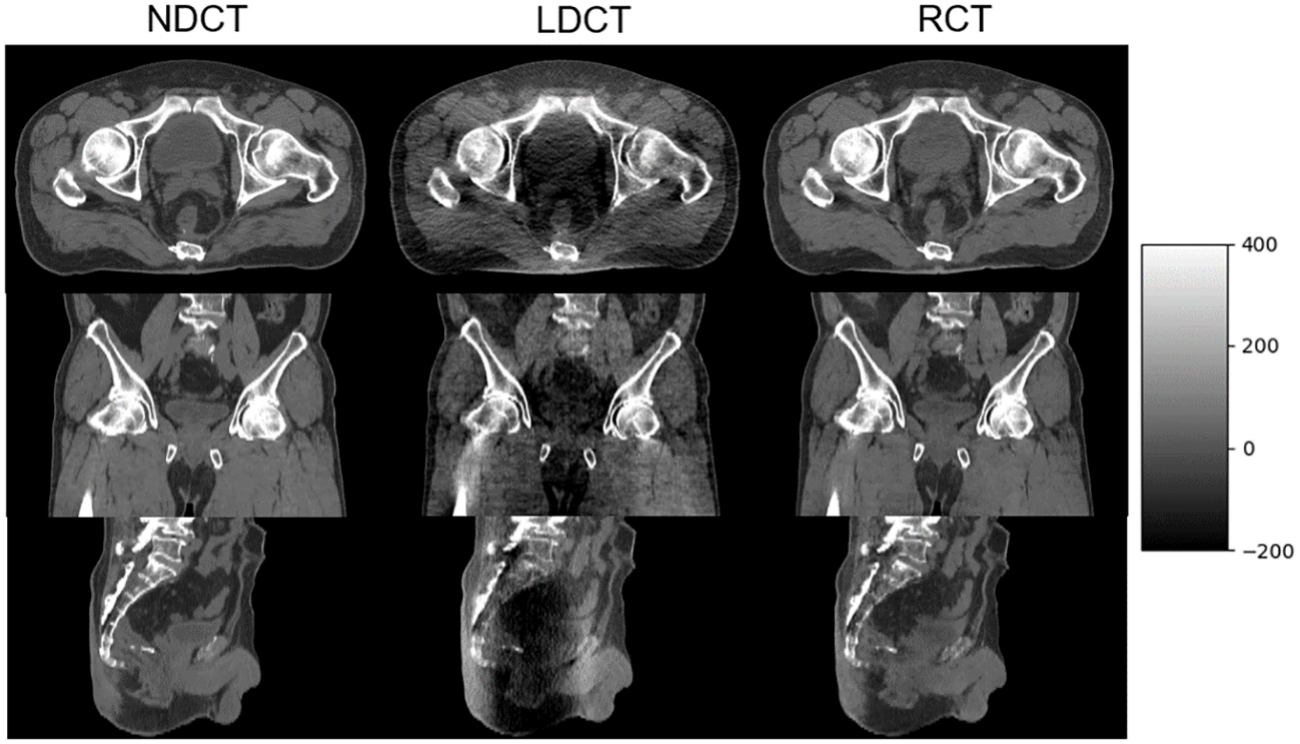
NDCT, LDCT, and RCT images from axial, coronal, and sagittal plane from one of the postoperative prostate cancer patients.

The axial, sagittal, and coronal slices of LDCT, RCT, and NDCT for one representative cases are shown in **Figure 7**. The image quality of RCT for all three planes show substantial improvement in terms of voxel values, spatial uniformity, and artifact suppression, compared to those of the original LDCT. When the tube current decreased, additional artifacts appeared in the middle due to the photon starvation effect. When the patient was overweight or has metal or other high-density materials (bones) in the body, the radiographic hunger effect may be more pronounced.

The mean and standard deviation for the HU of the four evaluation tissues in LDCT, RCT, and NDCT are summarized in **Table 1**. Obviously, the differences in average HU for the four evaluation tissues between RCT and NDCT were substantially suppressed compared to those between the original LDCT and NDCT.

**Table 1:** Mean and standard deviations of the Hounsfield Unit (HU) values of the regions of interest (ROIs) in the four evaluation tissues in LDCT, RCT, and NDCT.

		LDCT	RCT	NDCT
Muscle	Mean	46.80	44.05	43.85
	SD	1.86	4.8	4.75
Fat	Mean	−103.24	−144.57	−143.95
	SD	12.93	7.20	6.079
Bladder	Mean	19.02	−8.15	−4.45
	SD	5.98	6.06	11.62
Bone	Mean	222.86	246.76	256.85
	SD	29.74	24.03	22.43

**Figure 8:**
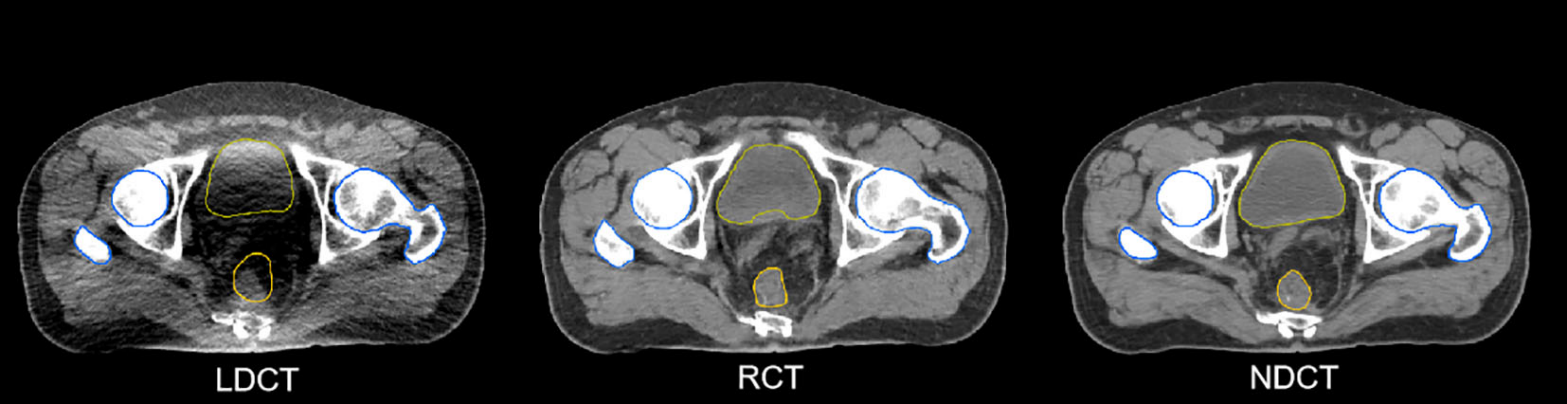
Different ROIs in LDCT, RCT, and NDCT.

The DSC results of each structure delineated by a radiation oncologist on the RCT, LDCT, and NDCT image are summarized in **Table 2**. The results showed that the DSC of the bladder and the femoral heads reached approximately 98%, and that of the CTV and rectum reached approximately 94%. The results indicated that the delineation results of target and OARs on RCT were consistent with that on the NDCT image, which met the clinical requirements. Compared with LDCT, the results of RCT had significant differences in the CTV, the bladder, and the rectum. The differences in ROIs are shown in **Figure 8**.

**Table 2:** Comparison of DSC results between RCT, LDCT, and NDCT.

	CTV	Bladder	The femoral head right	The femoral head left	Rectum
DSC (LDCT, NDCT)	0.79 ± 0.05	0.89 ± 0.04	0.97 ± 0.02	0.97 ± 0.02	0.61 ± 0.07
DSC (RCT, NDCT)	0.94 ± 0.02	0.98 ± 0.03	0.98 ± 0.01	0.98 ± 0.02	0.95 ± 0.06
*p*-value	<0.01	<0.01	>0.05	>0.05	<0.01

**Figure 9:**
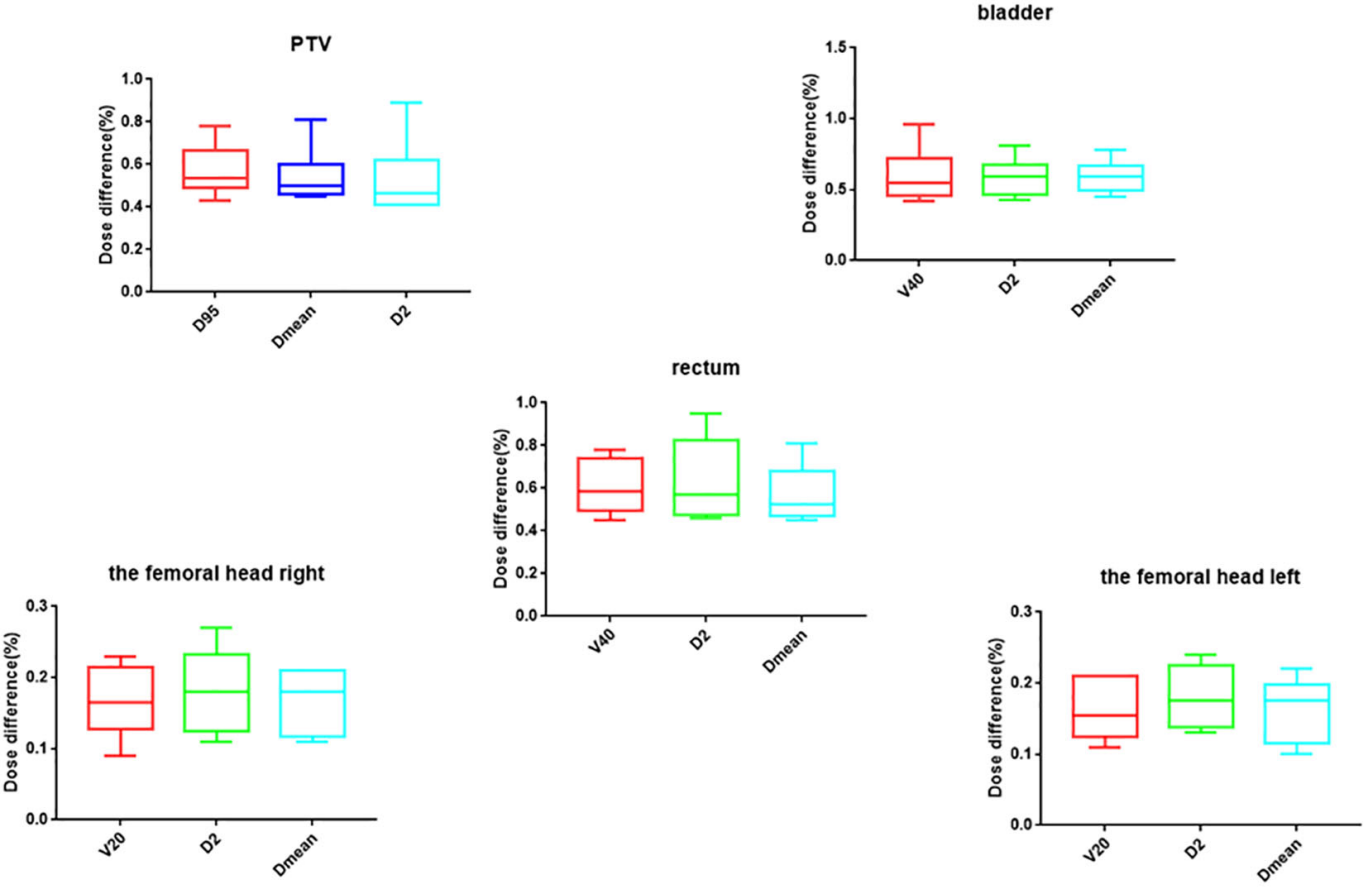
The dosimetric comparison of target and OAR between N_plan and R_plan.

The plan to perform ART on the NDCT was re-calculated on the RCT to verify the dose calculation accuracy of the RCT image. **Figure 9** shows the dose differences in the PTV, the rectum, the bladder, and the femoral heads between N_plan and R_plan. The results show that the differences in PTV on D_95%_, D_2%_, and D_mean_ are all within 1% between N_plan and R_plan. For the OARs, the dose differences of the femoral head right and the femoral head left are all within 0.5%. The differences of rectum and bladder are all within 1%.

## Discussion

Online-ART utilizing online image is a relatively new technique that is being rapidly implemented into clinical use to solve the former problem regarding target and organ deformation. The uRT-linac 506c linear accelerator integrates a diagnostic level FBCT, providing a high image quality online ART workflow. However, conducting ART in all 33 stages of postoperative prostate cancer radiotherapy will bring peripheral radiation dose to the patient. Frequent CT scans will increase the radiation dose to normal tissues of patients, thus increasing the risk of cancer. Therefore, we need to minimize radiation dose while ensuring high image quality for ART.

In this present study, additional doses of three image scanning methods, LDCT, NDCT, and MV-CT were measured to demonstrate the advantages of LDCT. A deep learning model of LDCT for abdominal and pelvic cancer patients was used to improve the image quality of LDCT scanned by reducing the tube current. In order to explore the feasibility of the RCT processed by the deep learning model in ART, the accuracy of delineation and dosimetry were compared between NDCT and RCT. The result showed that the image quality of RCT images was closer to that of NDCT. Compared with the NDCT, the DSC of target CTV reached 94%, and that of the normal tissue including bladder, femoral head, and rectum reached more than 95%. Compared with LDCT, the HU values of muscle, bone, bladder, and fat were greatly improved, and were closer with NDCT. There was no statistical significance in the dosimetry with the ART plan based on the RCT.

To the best of our knowledge, this is the first study to explore the feasibility of LDCT application in ART through deep learning, and compare the additional doses generated by different image scanning methods. The method proposed in this paper provides a powerful reference for online ART of LDCT, and can reduce the additional radiation exposure received by patients. However, there is no scientific evidence for LDCT images based on deep learning to be used for plan optimization at present. Therefore, the implementation of LDCT-based ART still needs to be explored and studied in subsequent clinical work. Another limitation of this study is that the training data require simultaneous collection of patients’ NDCT and LDCT. Patients need to sign an informed consent form, which limits the sample size of the training dataset. Can LDCT and localization CT used for daily image-guided radiotherapy be used as training data to extend the training dataset of the model? We will test the feasibility of this method in the future. In addition, the small size of patients for validation is also the limitation of this study. In the future, we will collect more data from a larger number of patients and a wider range of cancers to validate the potential of the proposed method in clinical applications.

## Conclusion

As IGRT and online ART are becoming increasingly prevalent in clinical use, they need to balance the risks associated with additional peripheral doses. In this study, a low-dose CT scanning for postoperative prostate cancer was performed by reducing the tube current of NDCT. A deep learning model of LDCT for the abdomen and pelvis was used to improve the image quality, and the peripheral dose of LDCT scanning to the patient was quantified and evaluated by a phantom and an ion chamber. Finally, in our opinion, it is feasible to use RCT images to achieve ART for postoperative prostate cancer and patients can benefit from the peripheral dose generated by image scanning compared with NDCT. In the future, we will collect more patient data to verify the feasibility of our proposed approach and develop online ART based on LDCT.

## Data availability statement

The original contributions presented in the study are included in the article. Further inquiries can be directed to the corresponding author. Requests to access the datasets should be directed to jh1215@foxmail.com.

## Ethics statement

The studies involving humans were approved by Medical Ethics Committee of the First Affiliated Hospital of Soochow University. The studies were conducted in accordance with the local legislation and institutional requirements. Written informed consent for participation was not required from the participants or the participants’ legal guardians/next of kin in accordance with the national legislation and institutional requirements. Written informed consent was obtained from the individual(s) for the publication of any potentially identifiable images or data included in this article.

## Author contributions

GG contributed to the conception and design of the study. JZ and WZ provided guidance. WG and HJ completed the data collection. LJ completes the network design and trains the network. GG drafted the manuscript and SW has made revisions to the manuscript. JZ supervises and manages the project. All authors contributed to the article and approved the submitted version.
